# An Interoperable Access Control Framework for Diverse IoT Platforms Based on OAuth and Role †

**DOI:** 10.3390/s19081884

**Published:** 2019-04-20

**Authors:** Se-Ra Oh, Young-Gab Kim, Sanghyun Cho

**Affiliations:** 1 Department of Computer and Information Security, Sejong University, Seoul 05006, Korea; terious551@sju.ac.kr; 2 Security Team, Naver Corporation, Bundang 13561, Korea; super.bg@navercorp.com

**Keywords:** IoT platform, access control, interoperability, OAuth 2.0, role, security requirements

## Abstract

Due to the rapid development of Internet of Things (IoT), IoT platforms that can provide common functions for things are becoming increasingly important. However, access control frameworks in diverse IoT platforms have been developed for individual security goals, designs, and technologies. In particular, current OAuth-based access control frameworks that are widely used in IoT research have not been providing interoperability among IoT platforms even though sharing resources and services is a critical issue for IoT platforms. Therefore, we analyze the main requirements for an IoT access control framework to properly design our framework and propose an interoperable access control framework based on OAuth 2.0 and Role. Our approach describes a new extended authorization grant flow to issue an Interoperable Access Token (IAT) that has a global access scope across IoT platforms using multiple pairs of clients’ credentials. With the IAT and proposed framework, we can access client-specific domains in heterogeneous IoT platforms, then valuable resources (e.g., data and services) in the domains can be accessed by validating the roles, which will greatly simplify permission management. Furthermore, IAT supports a simple token management (e.g., token issuance, refreshing, and revocation) by managing only one token for diverse IoT platforms. In addition, we implement our interoperable access control framework on Mobius and FIWARE, which are promising open-source IoT platforms, and test an interoperability scenario to demonstrate our approach with the implementation. Furthermore, the proposed framework is compared with other IoT access control approaches based on the selected requirements in this paper.

## 1. Introduction

The Internet of Things (IoT) is an environment in which things are connected to each other via the Internet and has been adopted as a promising technology by many companies such as Gartner, Cisco, and International Data Corporation (IDC). Consequently, global companies such as Google and Amazon have been researching and developing IoT-related objects (e.g., sensors and IoT devices) [1], which are expected to exceed 20 billion in 2020 [2,3]. In this context, IoT platforms and access control in the platforms are particularly important. The IoT platform will directly or indirectly affect connected things by providing common functions (e.g., communication, data management and security). If the strength of the access control in an IoT platform is insufficient, the things will be easily compromised by an attacker. Therefore, several IoT platforms such as Amazon Web Services (AWS) IoT, IBM Watson IoT and oneM2M have been developed with diverse access control approaches. However, the security responsibility is excessively distributed to each company since all IoT platforms are developed with their own security approaches, which have different attack surfaces and vulnerabilities. This poses a serious problem. The attack surfaces and vulnerabilities of each IoT platform will be merged during interworking, and studies [4,5,6,7] on the interworking between IoT platforms have actually been going on recently. Although OAuth-based access control frameworks have been widely researched to limit unauthorized access in the IoT field, secure interoperability among IoT platforms has not been properly considered. In fact, it is hard for conventional OAuth-based access control systems to interoperate with other OAuth-based systems since the existing OAuth 2.0 standard does not consider interoperability. To solve this problem, there is a need to extend the existing OAuth 2.0 standard for interoperability. Moreover, requirements for an access control framework in IoT environments should be analyzed based on the IoT characteristics and the existing access control framework research to properly design the interoperable access control framework.

Therefore, this paper analyzes the requirements for the proper design of an IoT access control framework and proposes an interoperable access control framework for heterogeneous IoT platforms based on OAuth 2.0 and role. Since most IoT platforms such as oneM2M and Future Internet ware (FIWARE) support REST APIs for resources, the OAuth 2.0 framework is used to limit an unauthorized access and the use of the REST APIs that can pose security issues in front of the framework. Moreover, role-based access control is applied for low-level resources (e.g., http://platform/domain/led/on), which are managed in client-specific domains. The administrator of a specific domain in an IoT platform can give a user privileges related to the low-level resources by assigning a role. This greatly simplifies the management of permissions [8]. In addition, we extend the existing grant flow in OAuth 2.0 framework to provide interoperability. Conventional Client Credentials authorization grant flow is used to issue an access token that has a local scope in a single domain since it only uses a single pair of client credentials that are assigned to a single domain. However, the extended authorization grant flow (i.e., Multiple Clients Credentials) issues an Interoperable Access Token (IAT) that has a global access scope across diverse IoT platforms using multiple pairs of clients’ credentials assigned in heterogeneous domains to access resources in the IoT platforms.

The main contributions of this paper are as follows:

(1) We analyze requirements for an access control framework in an IoT environment based on the characteristics of IoT (e.g., heterogeneity and resource constraints) and existing IoT access control research.
(2) Based on the analyzed requirements, we propose an interoperable access control framework using OAuth 2.0 that is straightforward and fast and Role, which simplifies permission management.
(3) We propose a new extended authorization grant flow to issue an IAT that has a global access scope across heterogeneous IoT platforms. With the IAT, various resources (e.g., data and services) can be easily shared among heterogeneous IoT platforms, and token management (e.g., token issuance, refreshing, and revocation) becomes easier since it can represent multiple numbers of conventional access tokens which have local access scopes for a single IoT platform.
(4) We demonstrate an interoperability scenario between the IoT platforms by implementing the proposed framework on Mobius and FIWARE, which are promising open-source IoT platforms.

The remainder of this paper is structured as follows: in Section 2, we analyze related works, and Section 3 describes target environment in which the proposed access control framework can be used as well as a role assignment request scenario proposed to mitigate scalability issue of roles. Section 4 presents an analysis of the 12 requirements for an access control framework in the IoT environment, as well as the proposed interoperable access control framework. In addition, interoperability scenario between IoT platforms is described and demonstrated in the Section 4. Finally, Section 5 compares the proposed framework with other IoT access control approaches based on the selected requirements, and Section 6 presents conclusions and future work.

## 2. Related Work

There are two studies on the basic security requirements and trends of IoT security standards. Oh and Kim [9] analyzed IoT security requirements based on three IoT characteristics (i.e., heterogeneity, resource constraints and dynamic environment) and six IoT elements (i.e., IoT platform, IoT network, service, attacker, user and cloud). Hwang and Kim [10] classified the security standards into six categories (i.e., confidentiality, integrity, availability, non-repudiation, authentication and authorization). According to their study, most security standards dealt with confidentiality, integrity, authentication, and authorization, and only few standards were focused on availability and non-repudiation.

IoT platforms often use OAuth 2.0 [11] to protect REST APIs, which provide resources and services. Authentication and Authorization for Constrained Environments (ACE) defined a lightweight authentication and authorization framework in an IoT environment based on Constrained Application Protocol (CoAP) and OAuth [12]. ACE also developed their own authentication, verification, encryption and signature methods in the application layer without existing technologies such as Transport Layer Security (TLS) and Datagram Transport Layer Security (DTLS). However, there are additional costs required to implement the new technologies in an existing environment. Although it is difficult to apply existing technologies such as TLS and DTLS to IoT, studies on TLS-based IoT security are still valuable, and researches are being conducted to enable the use of existing technologies in a constrained environment [13].

Srikanth et al. [14] described three scenarios to identify security threats (i.e., phishing, replay and impersonation attacks) that can occur while using OAuth. Sciancalepore et al. [15] proposed OAuth-IoT, a lightweight and flexible OAuth-based authentication and authorization framework designed for a constrained environment. In the literature, the features of three types of tokens (i.e., Bearer, JSON Web Token (JWT), and Proof-of-Possession (PoP) token) have been compared. Jung et al. [16] conducted research on implementing a personal OAuth authorization server on a user’s smartphone for an IoT environment. Authorization servers are generally external such as Google and Amazon servers; however, Jung assumed that an OAuth authorization server can be deployed in a user’s personal smartphone. The user can therefore control access. Solapurkar [17] and Fernandez et al. [18] also proposed OAuth-based security architectures for a secure automatic payment service and an access control model to enable application-scoped access control in an IoT environment. In particular, Solapurkar used JWT which includes digital signatures for third party authentication and described a detailed authorization process for the issuance of e-prescriptions in an IoT cloud scenario.

Access control in the IoT area is mainly based on Capability-based Access Control (CapBAC), RBAC and ABAC models and research has been conducted to utilize the advantages or complement the disadvantages of each model. Alramadhan et al. [19] studied several conventional access control models (e.g., Access Control List, RBAC and ABAC) used in IoT environments, and outlined the challenges (e.g., constrained resources and heterogeneity) for IoT access control. Ouaddah et al. [20] and Lee et al. [21] also investigated access control approaches in IoT environments, and proposed security goals (e.g., scalability, usability, flexibility, interoperability) to protect IoT security and privacy.

There have also been studies on IoT access control frameworks [5,22,23,24,25,26]. Sciancalepore et al. [5] proposed an IoT access control framework that takes into consideration heterogeneous IoT platforms based on Decentralized Multi-Authority Ciphertext-Policy Attribute-Based Encryption (DMA-CP-ABE) and ABAC. Furthermore, they described the targeted security requirements for the system (i.e., peer authentication, data confidentiality between communicating peers, access control based on cryptographic algorithms, user privacy, attributes with limited lifetime, revocation of attributes, and resilience against collusion attack). In addition, Pal et al. [22] proposed dynamic policy management and an IoT access control framework that can be used on constrained networks. One essential component of the framework is Central Management System (CMS), which is composed of a Role Manager (RM) and Capability Database (CD). To manage privileges in the access control framework, the RM manages roles and attributes, and the CD dynamically provides the capabilities of a user device to the RM. Bate et al. [23] analyzed IoT security objectives such as authentication, confidentiality, integrity and availability, and proposed an access control framework. In the framework, authentication between IoT devices and a hub is performed using a secure token issued by a cloud service, and authorization is decided based on authorization configurations and policies in an authorization server. Additionally, they proposed a combination of AES, Cipher Block Chaining (CBC) and Encrypted Salt-Sector Initialization Vector (ESSIV) for strong encryption of data stored in a resource directory. Andaloussi et al. [24] implemented Distributed CapBAC (DCapBAC) using JSON-based capability tokens and analyzed the pros and cons of three feasible IoT scenarios (i.e., centralized approach, distributed approach, and hybrid approach). Two other studies [25,26] were based on the ABAC model. Neto et al. [25] took advantage of a suite of cryptographic protocols to control access from unknown IoT devices with the consideration of the entire IoT device life-cycle (i.e., pre-deployment, ordering, deployment, functioning, and retirement). The access control framework proposed in [25] provides seamless interoperability with strong authentication for a new guest device. Ouechtati et al. [26] proposed an access control framework to integrate user behaviors and risk assessment in the ABAC model for enhanced access control in an IoT environment. The framework was also designed to be dynamically self-adapting to system failures, changes in resources and user needs.

## 3. Target Environment for the Proposed Access Control Framework

The proposed access control framework mainly limits unauthorized access based on OAuth 2.0. Since the proposed framework depends on HTTP to comply with conventional OAuth 2.0 specification, an IoT platform that deploys the framework should support HTTP. However, this limitation could be solved if the framework supports diverse protocols. In addition, the proposed framework uses a role to protect resources and simplify permissions management. The remainder of this section describes the target environment for the role-based access control.

**Figure 1:**
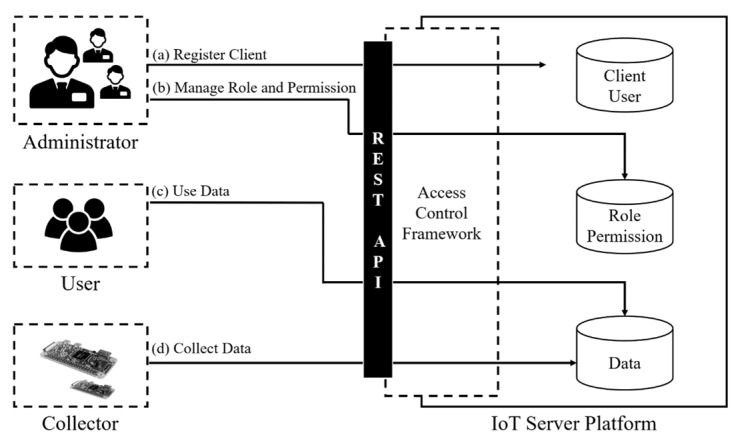
Common Scenario and Three Fundamental Roles in IoT.

Although IoT has diverse environments and scenarios, there are common roles. The main roles in an IoT environment are collector, user and administrator. The collector is a generic role for sensors, IoT devices, etc. which collect data. This role can be specialized in specific scenarios and has default permissions to access and write the collected data to a database or cloud. The user, on the other hand, retrieves, updates and deletes the data collected by the collector as well as other resources (e.g., services that a user created, and REST APIs provided by an IoT platform). Finally, the administrator manages specific domains. In a role-based access control model, the administrator defines the permissions for a resource, assigns the permissions to a role, then assigns the roles to a user. This provisioning step makes permission management very simple. For example, let’s assume there is a CCTV in a smart home. To keep the smart home secure, an administrator of the smart home domain could define a ‘family’ role that has the ‘checking CCTV’ permission, which can access videos in the smart home server, and assigns the ‘family’ role to a family member. In other words, the administrator can control the privileges of a user by assigning and unassigning the role. This method is more effective and efficient when the role consists of complex permissions. Without this kind of approach that simplifies permission management, permissions must be managed manually. For example, an access token that has a specific access scope should be reissued or the scope should be updated properly each time the related resources are updated. Figure 1 presents a common scenario and three fundamental roles in an IoT environment.

As shown in Figure 1, there are three fundamental roles (i.e., administrator, user and collector) in an IoT environment and they all have their basic functions (i.e., (a)~(d)). An administrator can register a client (e.g., home management system) with a redirection URI to an IoT server platform, and manage permissions and roles, which are dependent on the client. Once the provisioning is finished, a user and collector can request an access token from the access control framework using the authorization grant flows of the OAuth 2.0. In our approach, the proposed access control framework protects resources in an IoT server platform by restricting access in two phases.

**Figure 2:**
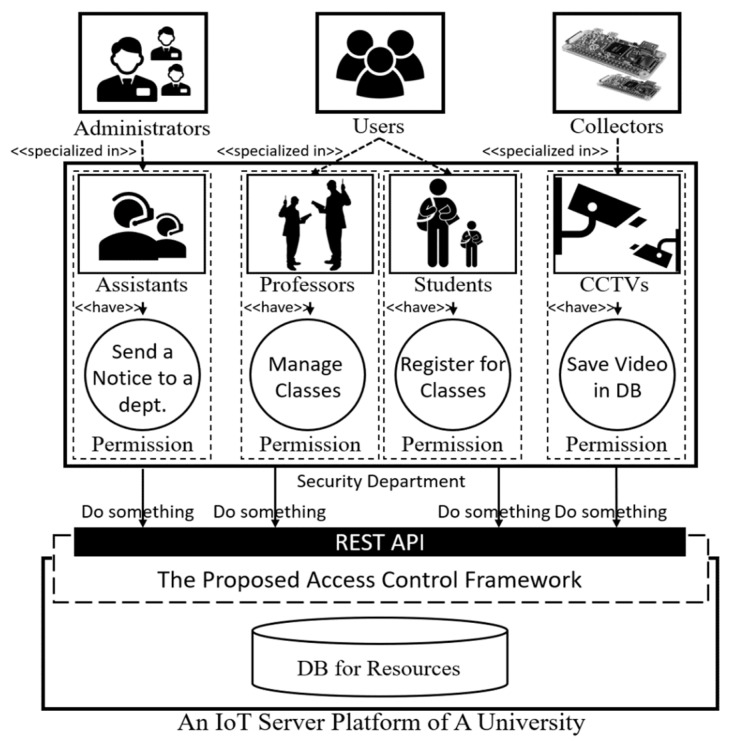
Simplified University Scenario with the Proposed Access Control Framework.

The first phase of the proposed framework limits access to high-level resources which is client-specific domains with an access token, and the second phase protects low-level resources (e.g., http://server/domain/*) managed in the client-specific domain based on the roles. The first phase is straightforward and fast when validating whether a requester has a valid access token to access a client-specific domain or not. However, as mentioned earlier, it is not appropriate for controlling access permissions for low-level resources since the structure of a low-level resource (e.g., http://server/hms/...) is more variable than that of a high-level resource (e.g., http://server/hms). Therefore, the proposed access control framework protects low-level resources with a role-based access control scheme that be used to manage an access scope by simply switching the role of the requester. Figure 2 shows a university scenario as an example of a target environment for the proposed access control framework.

In Figure 2, there are four roles (i.e., assistant, professor, student and CCTV) specialized from three fundamental roles (i.e., administrator, user and collector), and four permissions are assigned to each specialized role in the security department domain. As mentioned earlier, roles and permissions are managed domain-dependently, and if the subjects who have a role in the security department domain want to do something to the server, an access token for the domain and the appropriate roles are required. For example, only a subject with an access token for the security department and a professor role granted to manage classes can create a class for students using the REST API provided by an IoT server in the university. The target environment for the proposed framework could be a university, hospital, company, etc. The most important factor is that the target environments are mainly static environments. This means that once the roles and permissions are defined in a domain, they should not be changed frequently. Therefore, the proposed access control framework is difficult to implement in an ever-changing and large domain (e.g., smart city) since it is role-based. However, this does not mean the proposed framework cannot be adopted in an IoT environment because static environments are also part of the IoT. We will discuss this issue further in Section 5.

**Figure 3:**
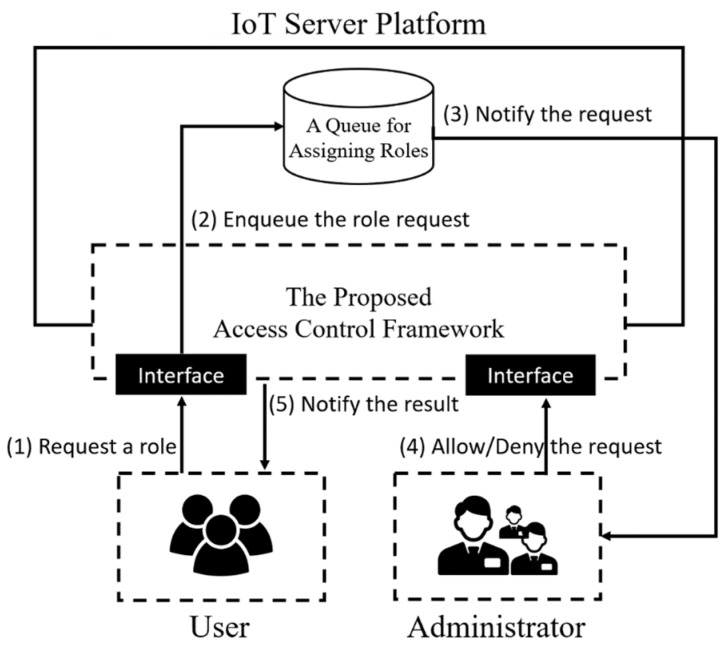
Role Assignment Request Scenario.

To mitigate scalability issues regarding the role, the proposed access control framework provides a role assignment request function as shown in Figure 3. In general, an administrator manually assigns a role to the user; however, a role assignment can be requested by a user through the proposed access control framework. To request a role, it is necessary for the user to be able to access the client-specific domain where the role belongs. If a user sends a role request to the specific domain using an access token and the token is successfully validated by the access control framework, the request will be placed in a queue with additional information on the requester such as IP and digital signature. Administrators in the given domain then receive a notification from the IoT server platform and decide whether the role request is appropriate or not. After that, the requester receives the decision result (i.e., allow or deny) as well as the requested role if the decision result is ‘Allow’.

Note that depending on the security level for the role, there may be roles that cannot be requested, and some roles can be assigned automatically without any intervention of the administrators according to pre-defined security policies. Further considerations in this regard are discussed in Section 5.

## 4. Proposed Interoperable Access Control Framework Based on OAuth and Role

### 4.1. Requirements for an Access Control Framework in an IoT Environment

In this subsection, we analyze 12 requirements for an access control framework in an IoT environment based on the main IoT characteristic (e.g., heterogeneity and resource constraint) and existing IoT access control researches mentioned in Section 2. The requirements are as follows.

- *Lightweight*: Things are more resource-constrained than conventional devices such as desktop computers and smartphones. Therefore, the security technologies applied to an IoT environment also need to be lighter. To reduce a burden of the things for authentication and authorization process, the proposed access control framework minimizes the load of sensors and IoT devices by delegating the authentication and authorization processes to the access control framework.
- *Flexibility*: An IoT access control framework should be able to authorize resource requests with flexibility. The proposed access control framework provides straightforward access control with OAuth 2.0 (i.e., access token) for high-level resources and flexible authorization for low-level resources based on the roles of a user.
- *Context-awareness*: The IoT contains diverse context information such as time, location, temperature and humidity in contrast with the conventional PC environment. If we use context information as a factor in the access control decision, better fine-grained access control can be supported. In order to support context-awareness, a requester accessing a particular resource (e.g., a service for locals) can send its context information to an IoT server platform, or the IoT server platform can verify its own context information (e.g., time on the server) based on the security policies of the resource when an access request comes in. The proposed framework supports context-aware access control by using the latter method (i.e., a server checks the context information by itself) and the security policies of a resource.
- *Scalability*: As the IoT environment is growing, an IoT access control framework should be scalable to handle the growing number of sensors, devices, roles, permissions, etc. The proposed framework is lightweight since it is token-based and the proposed role assignment request function improves the scalability by mitigating the need for role management. In addition, our proposed framework is less affected by the role explosion problem since we focus on a static domain.
- *Confidentiality*: To keep the user/client credentials and token secure, the communication must be encrypted. TLS and DTLS can be generally used for this purpose. Moreover, lightweight TLS (e.g., wolfSSL [27]), which is suitable for resource-constrained environments, was recently developed.
- *Integrity*: To ensure integrity, resources in an IoT server platform should only be controlled by legitimate users/clients. In our approach, a resource’s access authority is validated by an access token and roles. The integrity of data can be ensured with TLS, and the integrity of the tokens can be ensured using Hash-based Message Authentication Code (HMAC) in JWT.
- *Accountability*: The critical credentials (e.g., user/client credentials and access tokens) used in an access control framework should be audited. The proposed framework logs all access records to a resource and uses access tokens (i.e., records of token issuance, usage, expiration and refreshing) for accountability. However, the logging level can differ depending on the user’s security configuration level or the security policies of a resource and domain.
- *Privacy*: To protect privacy, data is managed in different client-specific domains, and only authorized users/clients can access the data. Personal access records on a resource are also related to privacy; however, the logging level is policy-dependent for each domain and resource. In contrast, access to the IoT server platform’s critical resources (e.g., security configuration file and user/client credentials) must be logged although privacy could be compromised.
- *Encryption/hashing for critical credentials*: User/Client credentials can be used to generate tokens; therefore, the credentials must be encrypted or hashed in database. Our proposed access control framework stores the credentials and access tokens in a hashed form in a database to prevent leakage.
- *Bypass prevention for access control framework*: Requests to an IoT server platform should be properly authenticated and authorized by the access control framework. No requests should be able to bypass the access control framework. The proposed access control framework performs security functions as a reverse proxy. In other words, the proposed framework intercepts all resource requests to prevent bypasses and checks whether the requester has legitimate authority to request a resource. If the requester has valid authority, the requested resource is forwarded from the proposed access control framework to the requester. Using this approach means that the resource server does not communicate with requesters that could be attackers.
- *Interoperability*: The proposed access control framework supports REST APIs for token issuance and access control. Since many IoT platforms such as FIWARE, oneM2M and ARTIK support RESTful interfaces, the proposed access control framework provides interoperability with other IoT platforms as well as legacy RESTful systems [18]. Furthermore, the proposed framework supports a new extended authorization grant flow to issue an IAT for simple sharing of valuable resources between IoT platforms.
- *Easy-to-use interfaces*: To support OAuth 2.0, the proposed access control framework provides a simple web server for managing users, clients, roles, permissions, etc. When using the web-based interface, web security (e.g., prevention of SQL injection) must be implemented.

Note that although the 12 requirements are important for an IoT access control framework, this research mainly focuses on the interoperability issue. Confidentiality, integrity, and encryption/hashing for critical credentials are guaranteed by existing mechanisms such as TLS and HMAC.

### 4.2. The Proposed Access Control Framework

**Figure 4:**
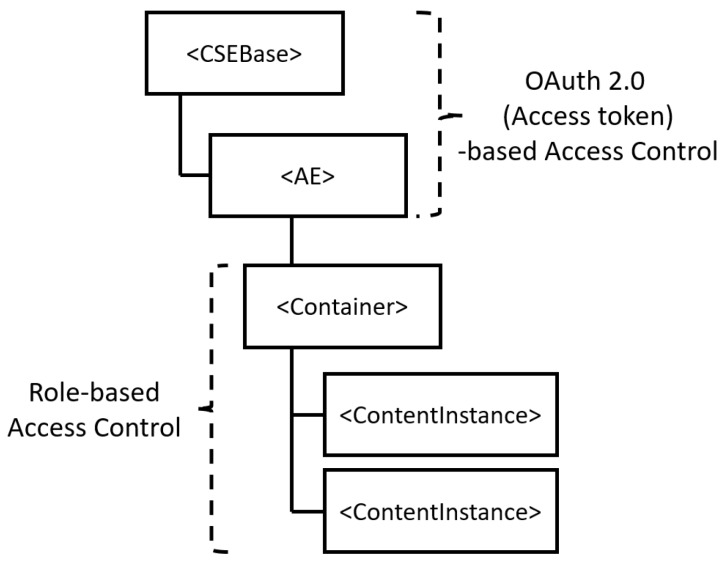
An Example Resource Tree and the Access Control Approaches in the Proposed Framework.

The proposed access control framework protects high-level resources (i.e., each client-specific domain) using the OAuth 2.0 access token, which is straightforward and fast. Low-level resources (i.e., resources in the specific domain), on the other hand, are accessed based on a role-based access control that makes permission management very simple. To highlight the difference between high-level access control and low-level access control, Figure 4 shows the access control approach for each resource level using the oneM2M resource tree as an example.

**Figure 5:**
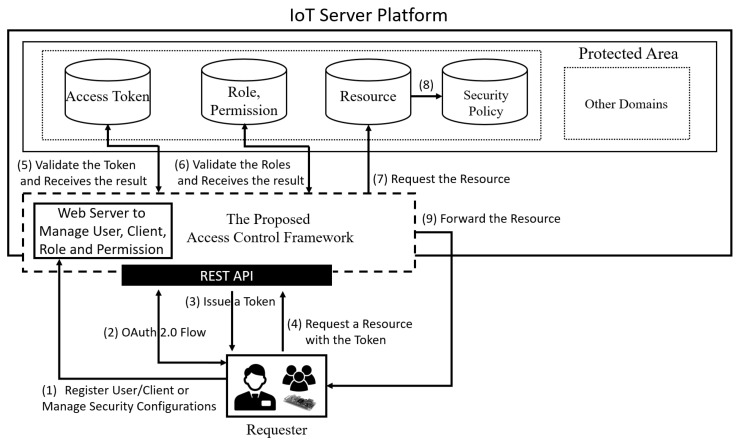
Entire Access Control Flowchart of the Proposed Framework.

As shown in Figure 4, access to a Common Service Entity (CSE), which represents a server, and an Application Entity (AE), which represents a client, in oneM2M is restricted by an access token issued through OAuth 2.0. Furthermore, access to a Container, ContentInstance and other lower level resources is limited based on the role of an access requester. For example, a Farm CSE could have a Barn Management AE consisting of a Light Container and a Light on/off ContentInstance. In this example, only a requester with a valid token issued for the Barn Management AE domain can access the Farm CSE and the Barn Management AE. On the other hand, the Light Container and the Light on/off ContentInstance can be accessed by a requester with a valid token and roles. An administrator of the Barn Management AE can manage the barn-related privileges (e.g., turn on the light in the barn) of a user by simply assigning and unassigning the roles. This approach, which enables simple permission management, is more useful when a low-level resource tree becomes deeper and more complex. Figure 5 shows the detailed flowchart of the proposed access control framework. In the figure, the proposed access control framework acts as an authorization server and the IoT server platform acts as a resource server. In other words, the proposed framework is separated from the IoT platform in a real environment and they communicate with each other through endpoints (e.g., using the introspection endpoint to validate an access token).

IoT server platforms support diverse REST APIs to provide resources (e.g., services and data). The proposed access control framework uses OAuth 2.0 and role-based access control to restrict the use of the REST APIs. The detailed flow is as follows:

(1) This is the provisioning phase. A requester registers the user and client information required for a token request. An administrator manages the permissions, roles, and security policies in this step. If the provisioning was already done, this step can be skipped.
(2) A requester initiates the OAuth 2.0 flow with the proposed access control framework using user and client credentials. If the requester has an access token, step two and three can be skipped.
(3) The requester receives an access token from the framework. The token is only used in specific client-specific domain (e.g., the Barn Management AE). In other words, the access token is managed client-dependently by using the scope of the token. Note that the scope is different in accordance with each design since there is no strict definition for the scope option in the OAuth 2.0 standard [11]. With our approach, the scope is a URL (e.g., http://farm/barn_management/) that represents the path of the resource.
(4) The requester accesses a protected resource (e.g., http://farm/barn_management/led/on) in the domain with the access token. Requests cannot bypass the framework. If the requester sends a resource request through other routes, the proposed access control framework should intercept the request.
(5) The proposed access control framework validates the token scope to determine whether the requester can access the requested client-specific domain or not. If the validation fails, the framework sends an error message to the requester, and the access control process is ended.
(6) If the requester is attempting to access a low-level resource corresponding to data in a specific domain (e.g., Container or ContentInstance in oneM2M), the framework validates the role assigned to the requester. If the requester wants to be assigned a specific role, the requester can request the role from an administrator as shown in Figure 3. If the validation fails, the framework sends an error message to the requester.
(7) If the role validation is successful, the framework forwards the resource request to the IoT server platform. The IoT server platform that receives the resource request retrieves the resource. However, the access control process is ended if there is no the requested resource.
(8) The IoT server platform also checks the security policies set on the requested resource. The security policies mainly describe the context-information. For example, a security policy can be set on a resource with an available time (e.g., time: 10:00–12:00 UTC).
(9) If all conditions required to use the resource are fulfilled, the IoT server platform sends the resource to the proposed access control framework, which then forwards the resource to the requester as a reverse proxy.

Basically, if a requester has an access token, an average of six steps (i.e., (4)–(9) in Figure 5) is needed to use a resource. For all steps, IoT things including sensors and IoT devices do not need high performance to be authenticated and authorized by the proposed access control framework since the access control processes are delegated to the framework. In addition, to ensure confidentiality, integrity and privacy for important data, TLS, DTLS or another lightweight TLS should be used. Access records to a resource and the token endpoint (e.g., token issuance and refreshing records) will be logged according to the security policies of the domain and resource.

**Figure 6:**
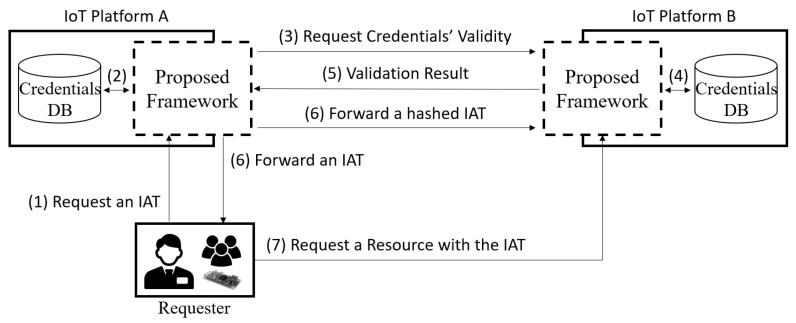
Flowchart Overview for Interoperability between Two IoT Platforms.

However, the process in Figure 5 only focuses on a single IoT platform. Although diverse IoT platforms have been developed and they each have their own valuable resources, existing OAuth-based systems cannot interoperate without additional processes since the OAuth 2.0 authorization framework standard does not consider interoperability. Therefore, our OAuth-based access control framework supports extended authorization grant flows that consider multiple domains. For example, Multiple Clients Credentials (MCC) that extends the existing Client Credentials grant flow uses multiple clients’ credentials to request an IAT that has a global scope across IoT platforms. Figure 6 shows an overview of the interoperability scenario between two IoT platforms using MCC. In this scenario, we assume that the IoT platforms A and B are trustworthy.

To easily share the valuable resources of trusted IoT platforms A and B, a requester can request an IAT through the proposed access control framework using extended authorization grant flows. Although the extended flows that mint an IAT have been designed and implemented in our proposed access control framework based on four existing authorization grant flows, this research only describes MCC because all grant flows basically have the same goal, which is the issuance of an IAT, and MCC is a very simple flow to describe and test the interoperability scenario. Section 5 describes some of the issues regarding extended authorization grant flows; the detailed flow and description of Figure 6 is as follows.

(1) A requester requests an IAT with a desired scope to the proposed access control framework using clients’ credentials. The client credentials consist of client_id and client_secret, so MCC generally needs several pairs of these. Client credentials can present the authority to access a client-specific domain; therefore, the requester can access as many client-specific domains as the pairs of credentials.
(2) The proposed framework in IoT platform A validates client credentials registered in A. If the client credentials are invalid, the requested scope associated with the client credentials is modified to limit access to the specific client domain.
(3) The proposed framework in A requests the validation of client credentials registered on IoT platform B since it cannot validate the client credentials stored in B by itself.
(4) The proposed framework in IoT platform B validates the client credentials provided by A.
(5) The proposed framework in B sends the validation result to A. The requested scope related to the client credentials could change according to the validation result.
(6) The proposed framework in A issues an IAT and then forwards the original IAT to the requester and forwards a hashed IAT to the B. By sharing the IAT with the requester and IoT platform B, the requester can use the original IAT to access a resource, and platform B can validate the IAT with the hashed IAT without any support from IoT platform A. In addition, a refresh token can also be issued in this step.
(7) Finally, the requester can request a resource related to the IAT’s scope from IoT platform B with the IAT issued from IoT platform A and it can request a resource from IoT platform A. Then, roles and security policies could check whether the requester can access the requested resource even though the figure does not describe this process in detail as Figure 5.

One of the extended authorization grant flows called MCC takes care of multiple pairs of clients’ credentials that represent access authorities for the client domains; therefore, based on the MCC, we can make an IAT that has a global scope across multiple IoT platforms to simply share resources. In the case of the existing OAuth-based access control framework, each token should be issued to access each resource in heterogeneous IoT platforms, and the token owner should manage all issued tokens. In other words, all tokens should be refreshed every time the tokens are expired, and also revoked when the tokens are no longer needed, or an attacker compromises the tokens. However, an IAT that represents the tokens which have local access scopes within a single IoT platform can simplify the token management. For example, if a refresh token for an IAT is issued in Step (6), a new IAT can be directly issued without additional processes (i.e., Steps (3)–(5) in Figure 6). On the other hand, the conventional way, which needs multiple tokens to access different domains, requires more requests for tokens’ issuance or refreshment. Furthermore, the existing OAuth-based IoT platforms (e.g., IBM Watson IoT, FIWARE, Oliot) must analyze the target platform’s access token format before translating token format of a specific IoT platform to one of the target IoT platform. It can be a big challenging problem if a lot of IoT platforms participate in interworking. However, unlike the existing approaches, the proposed framework does not need any additional translation processes.

### 4.3. Implementation

**Figure 7:**
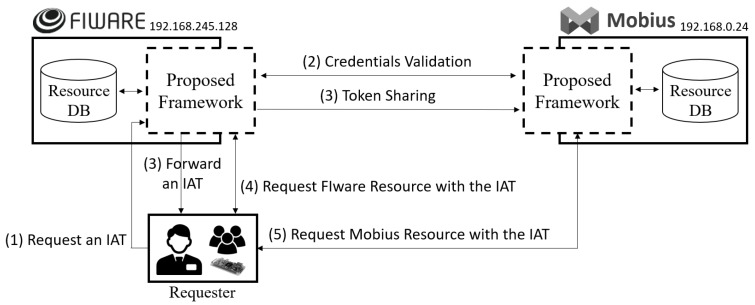
Interoperability Scenario between FIWARE and Mobius using an IAT.

We implemented the proposed access control framework on Mobius created by Korea Electronics Technology Institute (KETI) based on the oneM2M standard, and FIWARE, which consists of open-source IoT platforms on which we can test our proposed framework. The remainder of this subsection shows the detailed interworking process described in Figure 7.

**Figure 8:**
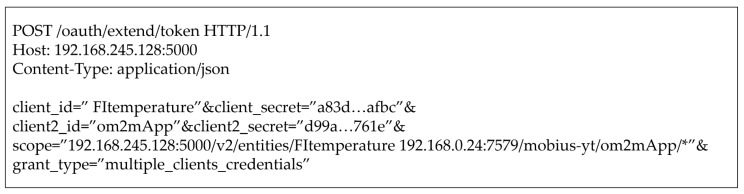
An IAT Request Format using MCC.

**Figure 9:**
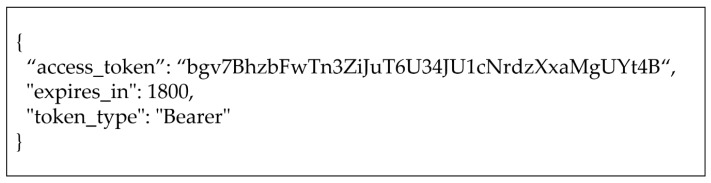
Forwarded Response from FIWARE to Requester.

**Figure 10:**
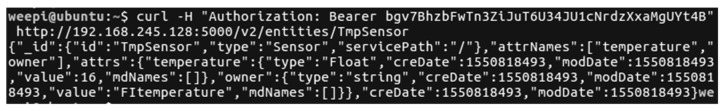
FIWARE Resource Request and Response using an IAT.

**Figure 11:**
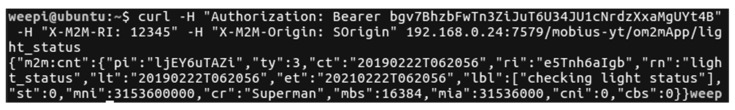
Mobius Resource Request and Response using an IAT.

This subsection assumes that all required provisioning steps (e.g., registering client) are satisfied in advance. The detailed flow for Figure 7 is as follows:

(1) The requester requests an IAT on the proposed framework (i.e., 192.168.245.128:5000) in FIWARE using MCC as described in Figure 8. As shown in the figure, two pairs of client credentials are used that are stored in FIWARE and Mobius. In this case, client 1 is registered in FIWARE, and client 2 is assigned in Mobius. The requester will be able to access the desired resources specified by the scope since the client credentials represent the access authority for the related resources. If the client credentials are correct, the requester can get the authority to access the resources.
(2) The proposed frameworks in FIWARE and Mobius validate client credentials and modify the scope if the credentials are invalid.
(3) The proposed framework forwards an IAT that has been created using the clients’ credentials to the requester and shares the hashed IAT with Mobius. If a refresh token is created, it should also be forwarded to simplify the token issuance process. Figure 9 shows the forwarded IAT from FIWARE to the requester. The response contains the access token, which is the IAT, the expiration time, and the token type.
(4) The requester requests a FIWARE resource (i.e., TmpSensor) with the IAT. The proposed access control framework in FIWARE checks the Authorization header to validate whether the token’s scope is valid for the requested resources. If the scope in the IAT is related to the requested resource, FIWARE retrieves the resource and forwards it to the requester as shown in Figure 10. If the IAT or scope of the IAT is invalid, FIWARE just sends an error message stating “Unauthorized.”
(5) The requester requests a Mobius resource (i.e., om2mApp/light_status) with the same IAT as is used to request an FIWARE resource. As in Step (4), if the scope of the IAT is related to the requested resource, Mobius retrieves the resource and forwards it to the requester as shown in Figure 11. If the IAT or scope of the IAT is invalid, Mobius sends an error message to the requester directly without any further validation of roles or security policies.

In this implementation section, we show the interoperability scenario between FIWARE and Mobius. By using the IAT, we can access heterogeneous IoT platforms without any token translation process, and token management becomes easier as mentioned in Section 4.2. Note that the token validation process between IoT platforms can be omitted if a refresh token is used to issue a new IAT.

## 5. Evaluations and Further Consideration

In this section, we compare the proposed IoT access control framework with other recent access control researches based on the selected security requirements and discuss several considerations. Note that some requirements (e.g., confidentiality and integrity) are removed from Table 1 since they are less focused on in this paper or are satisfied by other existing mechanisms such as TLS or HMAC, which are not our contributions. Table 1 shows a checklist for the considered requirements (i.e., lightweight, flexibility, context-awareness, scalability and interoperability) as well as the used access control approach.

Most studies in Table 1 considered several requirements well using conventional access control approaches such as ABAC, CapBAC, and RBAC. However, in the case of the interoperability, very few studies examined the interoperable access control framework for IoT platforms and no research has extended and implemented the OAuth 2.0 framework to make it interoperable for IoT platforms. As shown in Table 1, our proposed framework considered all selected requirements including the other requirements identified in Section 4.1. In addition, to prove our concept regarding the interoperability requirement, Section 4.3 described the detailed interoperability scenario between FIWARE and Mobius and implementation result. Even though the access control framework proposed by Neto et al. [25] in Table 1 satisfied all selected requirements like our framework, it did not focus on interoperability issue between heterogenous IoT platforms, but secure interoperability between new and guest devices.

Other considerations that have not been discussed are as follows:

- *Virtualization of the proposed access control framework.* The existing OAuth 2.0 framework does not consider interoperability. To provide interoperability between heterogeneous IoT platforms using the OAuth 2.0, we extended conventional authorization flows. However, common authorization capability and specification must be supported in heterogeneous IoT platforms. To this end, we could virtualize the proposed framework as an authorization layer. Once the authorization framework has been virtualized, IoT platforms can easily deploy the proposed access control framework and resources can then simply be shared by using the interoperable capability.
- *Extension of other existing authorization grant flows in the OAuth 2.0 standard.* We only used MCC in this paper to issue an IAT easily and focus on the proposed framework. However, other existing authorization grant flows should be extended since they have their own usage. Although this paper does not describe the other extended authorization grant flows, it will be specifically presented in future work.
- *Security consideration for the MCC and IAT.* The security of an original IAT and the multiple pairs of clients’ credentials used to issue an IAT are heavily dependent on TLS as the existing OAuth-based access control framework. In addition, if an attacker can intercept the hashed IAT while sharing the IAT step with other IoT platforms, it cannot be used since only the original IAT is required when the IAT has been validated. There could also be a security concern because of the IAT’s global scope, which means that a requester who has the IAT can access resources across multiple domains. However, critical low-level resources in client-specific domains are secured by role-based access control and additional security policies if an IAT is leaked. Any attacker who intercepts an IAT limitedly can access a resource that does not need any roles and security policies. For the security of existing parts of OAuth 2.0 framework, conventional security analysis documents could be referenced [11,28].
- *Security consideration for impersonation attack.* In MCC, a requester must send multiple pairs of credentials to get global access scope via heterogenous IoT platforms. However, one platform which received several pairs of credentials can impersonate the requester even though we assumed that IoT platforms are trustworthy. Therefore, the credentials should be encrypted or hashed so that only legitimate IoT platform can validate the credentials. For example, in order to obtain an IAT for global access across A and B, a requester sends its client credentials (i.e., pairs of client ID and secret of each IoT platforms A and B) to IoT platform A. And, client credentials for B would be encrypted with the public key of the IoT platform B to prevent the client credentials of IoT platform B from impersonation attack.
- *Regarding the role assignment.* A role is managed by an administrator of a client-specific domain. However, if there are administrators in a domain, there could be diverse criteria for managing a role. This could be a problem when the role is requested by a user. For example, let’s assume there are two administrators named Kim and Oh in domain A, and Kim knows a few users with malicious intent regarding a resource in the ‘my_client’ domain. In this context, if a user that Kim recognizes as an attacker requests a role and Oh grants the request, the user will compromise the resource. This is unlikely to happen if Kim sees the request earlier than Oh. Therefore, a role request should be granted when a certain number of administrators allow the request according to the security level of the resource. For a resource that requires a low security level, temporary roles can be assigned automatically when a user access the resource without the proper roles according to the security policies if the user has a valid access token for a client-specific domain.
- *Limitation of role-based access control approach and possibility of combination with ABAC.* Sensors and devices in an IoT environment have diverse attributes such as time, location, temperature, and humidity. As is well known, ABAC is used for fine-grained access control based on the attributes. We therefore can combine the benefits of the ABAC, which provides fine-grained control of access, with role-based access control, which can grant permissions by simply assigning/unassigning a role, to overcome the limitation of role-based access control approach.
- *Limitation of quantitative analysis for access control framework.* In this work, we did not conduct the quantitative comparison between the proposed framework and others. It is difficult to quantitatively evaluate the efficiency of the access control model because the model has different security policy each other. Furthermore, in many cases, most researches on access control framework do not deal with quantitative analysis and there are no common criteria for evaluation of the identified requirements (e.g., scalability and interoperability).

## 6. Conclusions

We have designed and implemented an access control framework that can be used in a static IoT environment. To properly design the IoT access control framework, we presented a common IoT scenario and target environment and analyzed 12 requirements of the IoT access control framework. The proposed access control approach uses OAuth 2.0 that protects the client-specific domain and role-based access control that protects resources in the domain. In particular, we extended the existing OAuth 2.0 to issue an IAT that has a global access scope across heterogeneous IoT platforms to easily share resources in the IoT platforms and simplify token management (e.g., token issuance, refreshing, and revocation). In addition, we implemented the proposed framework on Mobius, which is a oneM2M-based IoT platform, and FIWARE, which is a promising IoT platform in Europe. Then, we tested our interoperability scenario using an IAT based on the implementation result. The proposed framework quickly determined whether a user/client could access a given domain with a token and the administrators in the client-specific domain easily managed the role-related permissions. Finally, the proposed access control framework was compared with other IoT access control framework studies based on the selected requirements. There have been very few studies into an interoperable access control framework in IoT platforms and there has been no research into extending and implementing an OAuth 2.0 framework to make it interoperable with heterogeneous IoT platforms. In the near future, we will consider dynamic environments in IoT in addition to static environments. Access control frameworks in dynamic environments should be self-adapting and diverse real-time attributes should be considered dynamically for access-control decisions.

## Figures and Tables

**Table 1:** Comparison of Access Control Approach and Considered Requirements.

	AC Approach	LI	FL	CA	SC	IN	Remakes
Sciancalepore et al. [5]	ABAC	-	✔	✔	✔	✔	Consideration of the federation between heterogeneous IoT platforms
Sciancalepore et al. [15]	OAuth 2.0	✔	✔	-	✔	✔	Consideration of multiple token standards (i.e., Bearer, JWT, and PoP)
Fernandez et al. [18]	OAuth 2.0 and Role-based	✔	✔	-	✔	✔	Access control service is completely delegated to the server
Pal el al. [22]	Attribute-, Capability-, and Role-based	✔	✔	✔	✔	-	Access decision based on three features (i.e., attribute, capability, role)
Neto et al. [25]	ABAC	✔	✔	✔	✔	✔	Authentication and access control considering the entire life-cycle of IoT device
Ouechtati et al. [26]	ABAC	-	✔	✔	✔	-	Consideration of the subject behavior and the trust value
Proposed framework	Extended OAuth 2.0 and Role-based	✔	✔	✔	✔	✔	All requirements and interoperability between heterogenous IoT platforms are fully considered in the proposed access control framework

* AC: Access Control, LI: Lightweight, FL: Flexibility, CA: Context-awareness, SC: Scalability, IN: Interoperability.
